# ATP Release through Lysosomal Exocytosis from Peripheral Nerves: The Effect of Lysosomal Exocytosis on Peripheral Nerve Degeneration and Regeneration after Nerve Injury

**DOI:** 10.1155/2014/936891

**Published:** 2014-06-30

**Authors:** Junyang Jung, Hyun Woo Jo, Hyunseob Kwon, Na Young Jeong

**Affiliations:** ^1^Department of Anatomy and Neurobiology, School of Medicine, Biomedical Science Institution, Kyung Hee University, Hoegi-Dong 1, Dongdaemun-Gu, Seoul 130-701, Republic of Korea; ^2^Department of Pediatrics, Haeundae Paik Hospital, Inje University, 875 Haeun-daero, Haeundae-gu, Busan 612-896, Republic of Korea; ^3^Department of Anatomy and Cell Biology, College of Medicine, Dong-A University, Busan 602-714, Republic of Korea

## Abstract

Studies have shown that lysosomal activation increases in Schwann cells after nerve injury. Lysosomal activation is thought to promote the engulfment of myelin debris or fragments of injured axons in Schwann cells during Wallerian degeneration. However, a recent interpretation of lysosomal activation proposes a different view of the phenomenon. During Wallerian degeneration, lysosomes become secretory vesicles and are activated for lysosomal exocytosis. The lysosomal exocytosis triggers adenosine 5′-triphosphate (ATP) release from peripheral neurons and Schwann cells during Wallerian degeneration. Exocytosis is involved in demyelination and axonal degradation, which facilitate nerve regeneration following nerve degeneration. At this time, released ATP may affect the communication between cells in peripheral nerves. In this review, our description of the relationship between lysosomal exocytosis and Wallerian degeneration has implications for the understanding of peripheral nerve degenerative diseases and peripheral neuropathies, such as Charcot-Marie-Tooth disease or Guillain-Barré syndrome.

## 1. Introduction

Lysosomes are acidified, enzyme-containing intracellular organelles that break down phagocytosed materials, cell debris, and waste materials [1]. Therefore, lysosomes (conventional lysosomes) are considered to be the end-point of a final degradative pathway, the final destination of internalized macromolecules [2, 3]. However, it was recently demonstrated that lysosomes play an additional role in regulating exocytosis (secretory lysosomes) in addition to degrading old materials [4]: regulated secretion. This mature lysosome exocytic process can be triggered following an increase in the free Ca^2+^ concentration above 1 *μ*M. A microtubule-dependent step then provides the movement of exocytic lysosomes towards the plasma membrane [5]. Lysosomal vesicles are usually acidified by its H^+^-ATPase [4]. Chemicals that cause alkalinization of lysosomes can trigger lysosomal exocytosis [6].

Lysosomal exocytosis is required for plasma membrane repair via extracellular Ca^2+^ influx [7]. Plasma membrane resealing by lysosomal exocytosis is triggered within seconds after cell injury [7, 8]. Synaptotagmin VII, a plasma membrane Ca^2+^ sensor in lysosomal exocytosis, provides a mechanism by which a rise in intracellular Ca^2+^ upregulates the fusion of lysosomal vesicles with the plasma membrane [9, 10]. However, our understanding of the role of the lysosomal contents in the exocytic process of the peripheral nervous system (PNS) remains limited.

**Figure 1:**
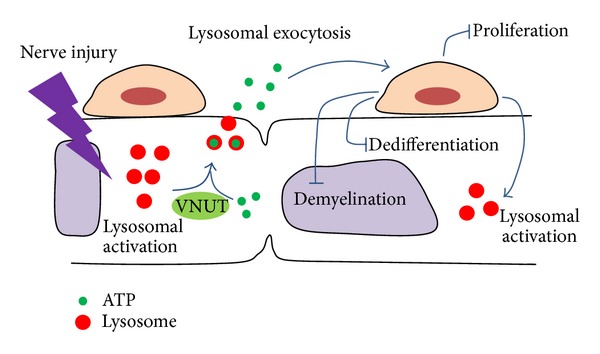
Model of lysosomal exocytosis events in Schwann cells during Wallerian degeneration. After peripheral nerve injury, secretory lysosomal activation is increased, which triggers lysosomal exocytosis during Wallerian degeneration. Through lysosomal exocytosis, Schwann cells release ATP into the extracellular space. The released ATP transmits to neighboring Schwann cells and promotes lysosomal activation and subsequent lysosomal exocytosis.

ATP is well established as a free energy source involved in biochemical pathways. However, ATP is now recognized as both an intracellular energy source and an extracellular messenger. Thus, ATP is a transmitter of relevant purinergic signaling in all nerves [11, 12]. In central synapses, there may be a corelease of ATP with other neurotransmitters or a separate release of ATP [13, 14]. ATP is a functionally important extracellular signaling molecule in the central nervous system (CNS) because activation of P2X and P2Y receptors in postsynaptic neurons, microglia, and astrocytes can trigger significant Ca^2+^ entry into the cytoplasm [15–17]. A recent study revealed that both resting microglia and activated microglia after nerve injury express P2X4, P2X7, and P2Y12 ATP receptors [18] and that released ATP contributes to the activation of the resting microglia near the activated microglia [19]. A previous report indicated that nonadrenergic, noncholinergic autonomic nerves contain ATP concentrated in lysosomal vesicles in vivo [20]. A considerable amount of ATP is stored and released by astrocytes and microglia through lysosomal exocytosis [21–24]. Contrary to a previous study [24], recently, it was reported that ATP release from microglia is dependent on the exocytosis via a vesicular nucleotide transporter (VNUT) but not lysosomal vesicles [25]. However, compared with glial cells in the CNS, the mechanism of ATP release via vesicular exocytosis in Schwann cells and peripheral nerve axons and their behaviors to Wallerian degeneration by released ATP in the PNS are not well known. Therefore, in this review, we discuss the dynamics of ATP related to lysosomal exocytosis in the PNS and the role of lysosomal exocytosis during Wallerian degeneration (Figure 1).

## 2. ATP Release through Lysosomal Exocytosis in the PNS

ATP is a significant signaling molecule in the PNS, as it plays an important role in chemical communication between several cell types [26, 27]. During Schwann cell development, extracellular ATP inhibits Schwann cell proliferation and differentiation [28]. In primary Schwann cells, extracellular ATP also triggers the release of ATP or amino acids [29, 30]. How can Schwann cells and peripheral neurons then release ATP into the extracellular space? One ATP-releasing mechanism in the PNS is secretory lysosomal exocytosis.

### 2.1. ATP Release from Schwann Cells through Lysosomal Exocytosis

In Schwann cells, uridine triphosphate or glutamate induces ATP release through vesicular exocytosis [31, 32]. Inhibitors of exocytosis that inhibit the formation of vesicles from the Golgi complex or prevent the delivery of vesicles disrupt ATP release from Schwann cells [31]. Recently, our group found that lysosomal vesicles are an exocytic ATP-releasing vesicle in Schwann cells [33]. Lysosomal-associated membrane protein 1 (LAMP1), a lysosomal vesicle marker, colocalizes with quinacrine, a specific ATP-combining chemical, in primary Schwann cell granules in culture, thus indicating that ATP is stored in lysosomal vesicles [33].

Fusion between exocytic vesicles and cell membranes is necessary to release vesicular contents. Vesicle-associated membrane protein 7 (VAMP7), a member of the vesicular soluble NSF attachment protein receptor (SNARE) family, is involved in Ca^2+^-dependent lysosomal exocytosis, and its interaction with synaptotagmin VII (SytVII), a member of the synaptotagmin family of Ca^2+^-binding proteins, is required for lysosomal exocytosis [34, 35]. In in vivo and in vitro Schwann cells, SytVII/VAMP7-positive vesicles are also observed in lysosomal vesicles [33]. The existence of SytVII and VAMP7 indicates that lysosomal exocytosis in Schwann cells is a Ca^2+^-dependent process.

VNUT has the capacity to transport cytosolic ATP into vesicles [36]. Intracellular vesicles that contain ATP through the interaction of VNUT are fused with the plasma membrane and, then, ATP through vesicular exocytosis is released into the extracellular space [36]. In Schwann cells, VNUT also induces the entry of ATP into lysosomal vesicles [33]. Thus, during Wallerian degeneration, VNUT induces ATP to enter lysosomal vesicles, and ATP is subsequently released through Ca^2+^-triggered lysosomal exocytosis in Schwann cells. These studies demonstrate that ATP is stored in lysosomal vesicles via VNUT following stimulation, and ATP secretion from Schwann cells occurs through Ca^2+^-dependent lysosomal exocytosis during Wallerian degeneration.

### 2.2. ATP Release from Peripheral Neurons via Lysosomal Exocytosis

ATP is liberated from stimulated peripheral nerves [37] and is important for signaling injurious nociceptive information [38]. ATP, as a neurotransmitter, is released from exocytic vesicles at presynaptic terminals and is the medium of the communication between the cells [28, 39–44]. In peripheral neurons, lysosomal vesicles contain a considerable amount of ATP in vivo [20]. However, the characteristics of ATP release via lysosomes in neurons remain to be elucidated. In dorsal root ganglion (DRG) neurons, the existence of lysosomal exocytosis and vesicular ATP release was reported separately [28, 43, 44]. Recently, our group reported that ATP is stored in lysosomes and is released from lysosomal exocytosis in cultured DRG neurons [45]. In primary DRG cultures, staining for quinacrine, an ATP-binding chemical, is visualized in lysosomal vesicles [45]. Quinacrine-positive vesicles were observed in neuronal soma and in the tip of elongating processes of cultured DRG neurons [45]. VNUT-positive vesicles containing quinacrine staining are also observed in neuronal soma and the tips of the elongating processes of cultured DRG neurons which are similar to the distribution of quinacrine-stained lysosomal vesicles [45]. Thus, in peripheral neurons, ATP was thought to enter into lysosomal vesicles through VNUT.

DRG neurons are pseudopolar neurons that contain both central and peripheral processes. Thus, ATP released from DRG neurons could affect DRG-glia interactions in the PNS and CNS. The existence of ATP-containing lysosomal vesicles in the tips of neuronal processes suggests the possibility that ATP released from DRG neuronal axon terminals through lysosomal exocytosis may induce microglial activation and neuropathic pain in the spinal cord dorsal horn after nerve injury [46], as well as Schwann cell proliferation or differentiation during Schwann cell development in peripheral nerves [28]. For example, lysosomal exocytosis is involved in axonal degradation during Wallerian degeneration. The high concentration of extracellular ATP, which is released from Schwann cells, inhibits axonal degradation in peripheral nerves during Wallerian degeneration [45]. Thus, neuronal mechanisms of ATP release through lysosomal exocytosis may increase our understanding of physiological or pathophysiological neuron-glia interactions in both PNS and CNS.

## 3. Lysosomal Exocytosis and Schwann Cell Demyelination

After nerve injury, during Wallerian degeneration, demyelination of Schwann cells occurs via fragmentation of the myelin sheath into ovoid-like structures near Schmidt-Lanterman incisures (SLI) [47–49]. Lysosomal activation is increased in Schwann cells during Wallerian degeneration [50, 51]. Increased lysosomal activation (conventional lysosomes), which indicates an increased number of acidified lysosomal vesicles, affects myelin fragmentation in Schwann cells during Wallerian degeneration [51]. It seems likely that the activated lysosomal vesicles engulf and remove myelin fragment debris in Schwann cells during Wallerian degeneration. However, the mechanisms by which the reduced lysosomal activation inhibits demyelination have not been studied previously. On the other hand, lysosomal exocytosis also occurs in Schwann cells during Wallerian degeneration. Recently, our group presented evidence that increased lysosomal exocytosis inhibits myelin fragmentation in Schwann cells during Wallerian degeneration [52]. Several lysosomal exocytosis activators (highly concentrated extracellular ATP and NH_4_Cl) inhibit myelin fragmentation in sciatic explant cultures during Wallerian degeneration [52]. In contrast, sciatic nerve explant incubation with both a lysosomal exocytosis activator and inhibitor (metformin and chlorpromazine) for 3 days restores myelin fragmentation [52]. Thus, we believe that one mechanism by which lysosomal exocytosis inhibits demyelination is through enhanced release of ATP from Schwann cells into the extracellular space in the PNS. The increased extracellular ATP level may induce Ca^2+^-dependent alkalization of existing acidified lysosomal vesicles in Schwann cells during Wallerian degeneration [24, 30] and may reduce the amount of activated conventional lysosomes. Decreased lysosomal vesicles may affect the inhibition of myelin fragmentation during Wallerian degeneration. In addition, the increased ATP concentration in the extracellular space may induce the alkalization of lysosomal vesicles and subsequently enhance lysosomal exocytosis in neighboring Schwann cells. In addition to the increased extracellular ATP, it is possible that unidentified secretory proteins induced by lysosomal exocytosis in Schwann cells prevent myelin fragmentation and degradation. Thus, further evaluation is needed to reveal the underlying proteins released by lysosomal exocytosis in demyelination during Wallerian degeneration.

During Wallerian degeneration, recruited macrophages into the peripheral nerves engulf the debris of myelin sheaths [53, 54]. Because macrophages express several ATP receptors [55, 56], the extracellular ATP may activate the recruited macrophages and, subsequently, inhibit the removal of myelin debris by the macrophage. However, because ex vivo Wallerian degeneration system is closed, the recruitment of macrophage into the sciatic nerve explants could be excluded. Thus, we think that the inhibition of demyelination in ex vivo sciatic nerves through the increased ATP concentration is not involved in the effect of macrophages [52].

Are there effects of lysosomal exocytosis during Wallerian degeneration other than ATP secretion in denervated Schwann cells? Lysosomal exocytosis is also involved in Schwann cell remyelination. Lysosomal vesicles in Schwann cells contain a compact myelin-consisting protein [57]. This secretory vesicle fuses with the plasma membrane through lysosomal exocytosis in Schwann cells and promotes remyelination by the addition of myelin protein to the plasma membrane [57]. Thus, several studies showed that lysosomal exocytosis in Schwann cells closely affects myelin sheath dynamics in response to stimuli.

## 4. Lysosomal Exocytosis and Schwann Cell Dedifferentiation and Proliferation

During Wallerian degeneration, Schwann cells detached from axons undergo dedifferentiation and reenter the cell cycle to promote axonal regeneration. These dedifferentiated Schwann cells are similar to their immature state during Schwann cell development. The transition from myelinating Schwann cells to their dedifferentiated state involves several regulatory proteins. Extracellular signal-regulated kinase (ERK), c-jun, and p38 mitogen-activated protein kinase (p38 MAPK), members of the MAPK family, induce the initiation of Schwann cell dedifferentiation and act as negative regulators of myelin differentiation in Schwann cells [58–61]. The p75 neurotrophin receptor (NGFR), which is a low affinity nerve growth factor receptor, is activated in demyelinating myelinated Schwann cells after nerve injury and is involved in the Schwann cell dedifferentiation process during Wallerian degeneration [50, 51, 62]. p75NGFR induction also mediates lysosomal activation in demyelinating Schwann cells during Wallerian degeneration [51]. Thus, understanding the relationship between lysosomal vesicles and members of the MAPK family or neurotrophin receptors during Wallerian degeneration may help to identify the molecular mechanism of Schwann cell dedifferentiation.

Using a sciatic nerve explant system, our group found that p38 MAPK and ERK1/2 are involved in lysosomal exocytosis in Schwann cell dedifferentiation during Wallerian degeneration [63]. A lysosomal exocytosis activator (i.e., highly concentrated ATP) induces the downregulation of p-p38MAPK and p-ERK1/2 in Schwann cells during Wallerian degeneration [63]. Lysosomal exocytosis is involved in p75NGFR expression and lysosomal activation during Wallerian degeneration. Highly concentrated ATP (2 mM) inhibits conventional lysosomal activation and the expression of p75NGFR in the denervated state of Schwann cells during Wallerian degeneration [63]. At this time, a decrease in conventional lysosomal activation is likely to induce the reduction in acidified vesicles for scavenging myelin fragments and the transfiguration into secretory vesicles (secretory lysosomal activation). Thus, these studies indicate that lysosomal exocytosis affects Schwann cell dedifferentiation during Wallerian degeneration. In addition, increased lysosomal exocytosis blocks Schwann cell proliferation, which is involved in axonal regeneration during the process of peripheral nerve regeneration [63].

Which molecules released through lysosomal exocytosis affect Schwann cell dedifferentiation during Wallerian degeneration? During Schwann cell development, increased extracellular ATP (300 *μ*M) inhibits the proliferation and differentiation of Schwann cells cocultured with DRG neurons [28, 64]. Because Schwann cells return to an immature developmental stage during Wallerian degeneration, it is possible that ATP released through lysosomal exocytosis could affect dedifferentiating Schwann cells during Wallerian degeneration. ATP released from dedifferentiated Schwann cells during Wallerian degeneration may function as a neurotransmitter in the peripheral nervous system and communicate with neighboring Schwann cells to inhibit their dedifferentiation. Thus, these studies have confirmed that ATP and lysosomal exocytosis in the PNS are closely related to Wallerian degeneration.

## 5. Concluding Remarks

The role of lysosomal exocytosis in the PNS has been studied recently. The current belief is that lysosomal exocytosis is involved in Schwann cell demyelination, remyelination, dedifferentiation, and proliferation during Wallerian degeneration. In addition, secretory vesicles affect axonal degeneration during Wallerian degeneration. In the PNS, an important role for lysosomal exocytosis is that it releases ATP from peripheral neurons and Schwann cells. ATP may function as a neurotransmitter and affect nerve degeneration during Wallerian degeneration. According to our previous studies [52, 63], peripheral nerve injury should increase ATP release through lysosomal exocytosis into the extracellular space of the sciatic nerves and the increased ATP should have inhibited Wallerian degeneration in the injured sciatic nerves without any treatments of lysosomal exocytosis activators. However, Wallerian degeneration in the injured sciatic nerves without any treatments is ongoing [52]. Then why does not the inhibition of Schwann cell dedifferentiation and proliferation through secretory lysosomal ATP release occur in vivo during Wallerian degeneration? We believe that extracellular ATP released from Schwann cells or peripheral axon terminals is easily degraded in the extracellular environment in vivo [65]. The efficient prevention of ATP degradation in the extracellular space is likely to regulate the processes of Schwann cell dedifferentiation and proliferation during Wallerian degeneration. Consequently, these recent results have opened up a new research area to understand the mechanisms of peripheral nerve degeneration and regeneration. Furthermore, the regulation of ATP release in peripheral nerves may make lysosomal exocytosis a potentially valuable therapeutic target for peripheral nerve degenerative diseases and peripheral neuropathies, such as Charcot-Marie-Tooth disease or Guillain-Barré syndrome.
